# Editorial Items

**Published:** 1861-01

**Authors:** 


### EDITORIAL.

*Delay of the January Number Explained.*—We regret that the present No. has been delayed by unforeseen circumstances. The gentleman with whom we had made a verbal contract to print the *Journal* the ensuing year, disappointed us in the very nick of time, and the necessity of making entirely new arrangements caused a more provoking, because wholly unanticipated, delay. The February No. will be issued early in the month, and thereafter our Printers contract to mail each No. promptly at a period not later than the first day of the month designated on the *Journal.*

---

*The Future.*—The Editors beg leave to say that for the ensuing year they have perfected arrangements which, it is believed, will add largely to the interest, importance, and moreover the artistic appearance, of the Journal committed to their charge. Original contributions, of both scientific and practical character, have been guaranteed from gentlemen of eminence and real worth in the profession; and the several departments of Selected Reading, Abstracts, Reviews, Bibliographical Notices, Items of Medical News, &c., &c., it is intended shall be a true reflex of medical thought, science, art and advancement.

In this, the *Eighteenth Volume* of the Journal, enjoying as it does pre-eminently, the largest and widest circulation of any medical periodical in the Northwest, the Editors hold it as incumbent upon them to use their best efforts to render it even more worthy than ever of professional support and influence. Disclaiming the character of an *organ*, in the conventional sense of that term, it will still attempt to be an *exponent* of sound medical opinion, whether in reference to professional literature or education, science or art. It aims to be a welcome guest at the tables of the faculty of the Northwest, as well as to indicate to more remote portions of country the professional *status* of its immediate section. Little space will be granted to subjects of medical foibles or follies, within or without the professional ranks, whilst the Journal will still claim the right to independent discussion of real problems of interest, and an earnest outspoken defence of what it believes to be the right and the true, amid all the variety of topics which can profitably claim professional attention.

---

*To Contributors.*—With sincere thanks to the many friends of the Journal who have heretofore added so much to its interest and value by their generous contributions to its pages, we hopefully solicit a continuance of their favors in the future. It is important, in order to secure early insertion, that articles should be sent in before the *fifteenth of the month.*

Brevity, perspicuity and precision, are the necessary elements of a first-rate medical communication. Rhetoric is better adapted to ornament secular than medical magazines, even when in the latter so exciting a subject as *inversio uteri* is to be ventilated.

---

*Rush Med. College.*—About 160 gentlemen are in constant attendance upon the present annual session of this institution. Of this number 145 are matriculated and have taken out all the tickets.

---

*Summer Session of Rush Medical College.*—The special attention of medical students is invited to the announcement of this course, to be found on the cover. The high character and excellent professional attainments of the gentlemen having this institution in charge, would be sufficient to secure its success, even if previous sessions had not already demonstrated the fact. We lend the enterprise hearty endorsement and support.

*Surgical Instruments.*—The enterprising and popular house of J. H. Reed & Co., of this city, send out with this No. a full catalogue of their stock of surgical instruments, accompanied with the further convenience of a list of prices. It is worth preserving for future reference.

*Per-Sulphate of Iron in Post-Partum Hemorrhage.*—Dr. Geo. Mendenhall, of Cincinnati, reports a case of successful treatment of dangerous hemorrhage by intra-uterine injection of three ounces of the saturated solution of per-sulphate of iron in water. All the usual remedies had failed, and the case seemed desperate. The effect was prompt and complete. "*After the injection there was not at any time a particle of fresh blood, or a tinge of it, in the lochia.*"

*Adulteration of Alcoholic Spirits.*—Dr. A. A. Hayes, State Assayer of Massachusetts, as the result of considerable experimentation, doubts the intentional adulteration of alcoholic spirits by strychnine or other foreign poisons. He considers the spirits produced in this country to serve as beverages, remarkable for their purity and freedom from any substances which careful rectification can remove. The low-priced common spirits are occasionally noxious from the presence of the so-called *fusel oils.* But salts of copper, of lead or tin, are not infrequently present in newly distilled spirits, derived from the condensers in which the vapors are reduced to a fluid form. The quantity, however, is necessarily small, and mainly important in toxicological analyses when death is suspected to have resulted from poisons derived from other sources.

*The St. Louis Medical and Surgical Journal* for November, contains a severe attack upon Dr. Jos. N. McDowell's Report on Surgery. We regret to see the unpleasant feeling manifested, but believe the parties will respectively survive.

Dr. Linton winds up his series of articles on Homœopathy, having in the most trenchant, unanswerable, and yet genial, style, annihilated his *quasi* antagonist. By the by,—this is one of the most readable of our exchanges.

---

*Gonorrhœa.*—Some one lately revives the medicated bougie in the treatment of obstinate cases of gonorhœa—the wax bougie encrusted with pulv. alum, being recommended. It is to be retained in the urethra several hours at a time. This is well enough, perhaps; but the real secret of the successful treatment of this disease is merely this: to secure the application of the medicament to the diseased surface, a thing rarely done, and hence the frequent failures. The poisonous matter finds lodgment in the crypts, the lacunæ, of the urethral parietes, and in old cases not infrequently these become largely distended. Obliterate these, by patient distension of the passage, so that the antidotal medicine may be applied directly to the diseased part, and the difficulty is ended. The methods are obvious, and success about infallible.

---

*Seventeenth Semi-Annual Report of the Board of Water Commissioners*, 1860.—The city of Chicago boasts the purest water, plentifully distributed, of any city of its size. The wonderfully augmented salubrity of the city for several years past, is unquestionably in great part due to the purity of the water with which it is supplied, and the improved system of sewerage which has been inaugurated and carried forward with commendable dispatch. Let the good work go on.

---

At a recent meeting of the Medico-Chirurgical Society, of New York city, Dr. A. B. Mott stated that he had employed Prof. Brainard's method of subcutaneous division of the bones and soft parts in ununited fracture several times with success. —*Med. and Surg. Jour., Nov.* 24, 1860

---

*Nicotian.*—It is estimated that the world smokes, snuffs and chews 2,000,000 tons of tobacco annually.

*Palliative Treatment of Cancers.*—Mr. Thos. Hunt, (Southern M. and S. Journal), thinks much can be done in the palliative, and perhaps curative, treatment of "those cases of true scirrhusin the breast in which there is a hard and movable tumor, not yet advanced to the stage of ulceration." He recommends the belladonna plaster to allay pain ; cotton wadding to protect from changes of temperature ; suspension of the whole breast especially if pendulous ; generous diet, tonics, particularly chaly beates and arsenic ; moderate but frequent exercise in the open air, &c., &c. The course recommended seems eminently judicious.

---

*Climate of California in Phthisis.*—Dr. James Blake, of Sacramento, eulogizes the climate of California for Consumptives, in a series of well written articles in the Pacific M. and S. Journal. At the present time the attention of practitioners seems to be directed about equally to California and Lake Superior as resorts for invalids of this character. So much depends upon the peculiarities of each particular case, that it would seem difficult or impossible to lay down any positive rule for general adoption.

---

*Proceedings of the American Institute of Homeopathy, Philadelphia*, 1860, *pp.* 206.—From careful reading of this pamphlet, the idea, which had previously gained possession of our minds, that the disciples of Hahnemann were gradually dropping the more ludicrous and narrow features of their creed, becomes further confirmed. In general practice, it is notorious, that the gentlemen who label themselves Homeopathists, do not scruple to avail themselves of the appreciable doses, and most of the later books and pamphlets emanating from that school, are deeply tinged with the same heresy against their Grandfather's dogmas. The *let-alone* treatment will result in speedy convalescence to the victims of the infinitesimal epidemic.

*Quien Sabe?—Grease an Antidote for Arsenic.*—Blondlot shows by experiment that grease reduces the solubility of arsenious acid to one-fifteenth or twentieth its previous degree. "Morgagnie tells us, in his writings, that in his time, the Italian boatmen used to astonish the bystanders by swallowing, without hurt, large pinches of arsenious acid, having taken the precaution beforehand, of drinking a quantity of milk, or eating some greasy matter. As soon as the public retired, they got rid of the poison by vomiting." This would seem to indicate the use of cream, lard, &c., as antidotal to this poison. The remedy proposed, at all events, has the advantage of being innocuous.

---

*Ligature of the Arteria Innominata.*—Dr. E. S. Cooper, of San Francisco, reports a recent case of ligation of the *arteria innominata.* Thirty days from the operation the case was progressing favorably, although from the final results of recorded cases of the kind, he was still watching for the sequel with considerable anxiety. One thing is quite evident, from the remarkably favorable results chronicled by our brethren of the Pacific coast, and that is, that the climate of their section is peculiarly salutiferous to surgical patients.

---

*Monsel's Salt.*—Our inquiring friend is informed that by this term is meant the per-sulphate of iron, a remedy recently grown very popular as a styptic, and also as a local application to venereal and other ulcers.

We find the following formula from the Am. Jour. Pharmacy a good one: By George S. Dickey, Jr., of San Francisco, Cal.—I give you my formula, the result of numerous experiments when I first undertook its manufacture:

| | | |
|---|---|---|
| ℞. | Aquæ destillatæ, | ℥ lxxx. |
| | Acid Sulph. Com. | f. ℥ ix,Xf ʒ iii. |
| | Ferri Sulph. Puræ, | ℥ c. troy. |
| | Acid, Nitric, " | f. ℥ viii. or q. s. |

Mix the water and sulph. acid, and dissolve in the mixture one-half of the sulphate of iron with the aid of heat. Bring up the mixture to a brisk boil, and add the nitric acid little at a time until effervescence ceases, and while still boiling add the remainder of the sulphate of iron little by little, and boil until effervescence ceases. Filter the solution, evaporate to a syrupy consistence and spread on plates of glass to dry. It requires considerable heat to dry perfectly, but is quickly dehydrated by a too long continued heat. When dry, it is necessary to detach it from the plates with a chisel.

The article has attained great celebrity here, principally as a hæmostatic and as a local application to venereal ulcers.

I have manufactured and sold more than two thousand (2,000) ounces during the last year and a half, and its sale still continues, in fact, increases.

I have never yet met an instance of its failure to stop bleeding when properly applied, and it is only necessary that the dry salt should be sprinkled on the wound.

I have been much surprised that it has been so long getting into use in your section, as I sent two or three samples to different parties near a year since.

Very truly yours, GEO. S. DICKEY, JR.*

### THE AMERICAN MEDICAL ASSOCIATION.

The *Louisville Medical News*, in its penultimate number, is exercised with reference to Chicago and the ensuing meeting of the American Medical Association. We appreciate its depth and tenderness of emotion. We know that when that august body was about to convene at Louisville there were local wars and rumors of wars, log-rolling and feastings, and cliqueing in abundance. We do not wonder that the Louisville editor thinks the same is the case in Chicago. Some people are very apt to reason from particulars to generals, on a very small allowance of particulars. We beg leave, however, to assure our chivalrous *confrere* that, as yet, we have not heard the subject alluded to in this city, publicly or pri-

*The specimens of the salt received from Mr. Dickey were the finest we have seen; perfectly dry, in very thin scales, translucent and of a light reddish brown color, very soluble and astringent.—ED. AM. J. PH.

vately. The only medical gentleman who seemed to manifest any interest in the matter, has in a contemporaneous print disavowed any ambition in the premises, and from sundry circumstances we are inclined to credit his disavowal.

The profession in Chicago we believe will sustain the reputation of the city for hospitality and good cheer, next June as heretofore.

The political squabbles and eccentric movements which a few demagogues are howling about, just at this period, we do not believe will materially influence the annual assemblage of AMERICAN physicians. Medicine is cosmopolitan, and is not likely to prostitute itself before sectional ululation, whether Northern or Southern. Time was when a venerable fragment of Philadelphia excreta, could rail at western medical men, and assert that wise men came only from the east, but just now the professional sense has opened itself to the idea that true medical science knows no South, North, East or West, but limits itself only to the round world and its circumambient atmosphere. This is true—Chicago says to the American profession —we have no friends to reward or enemies to punish. If you have any honor to confer, *Detur digniori*, irrespective of geography or chicane.

